# Selection of Newly Identified Growth-Promoting Archaea *Haloferax Species* With a Potential Action on Cobalt Resistance in Maize Plants

**DOI:** 10.3389/fpls.2022.872654

**Published:** 2022-05-19

**Authors:** Samy Selim, Nosheen Akhtar, Nashwa Hagagy, Awadh Alanazi, Mona Warrad, Eman El Azab, Mohammed Yagoub Mohammed Elamir, Mohammad M. Al-Sanea, Soad K. Al Jaouni, Mohamed Abdel-Mawgoud, Anis Ali Shah, Hamada Abdelgawad

**Affiliations:** ^1^Department of Clinical Laboratory Sciences, College of Applied Medical Sciences, Jouf University, Sakaka, Saudi Arabia; ^2^Department of Biological Sciences, National University of Medical Sciences, Rawalpindi, Pakistan; ^3^Department of Biology, College of Science and Arts at Khulis, University of Jeddah, Jeddah, Saudi Arabia; ^4^Botany and Microbiology Department, Faculty of Science, Suez Canal University, Ismailia, Egypt; ^5^Department of Clinical Laboratory Sciences, College of Applied Medical Sciences at Al-Quriat, Jouf University, Al-Quriat, Saudi Arabia; ^6^Pharmaceutical Chemistry Department, College of Pharmacy, Jouf University, Sakaka, Saudi Arabia; ^7^Hematology/Pediatric Oncology, Yousef Abdulatif Jameel Scientific Chair of Prophetic Medicine Application, Faculty of Medicine, King Abdulaziz University, Jeddah, Saudi Arabia; ^8^Department of Medicinal and Aromatic Plants, Desert Research Centre, Cairo, Egypt; ^9^Department of Botany, Division of Science and Technology, University of Education, Lahore, Pakistan; ^10^Integrated Molecular Plant Physiology Research, Department of Biology, University of Antwerp, Antwerpen, Belgium; ^11^Botany and Microbiology Department, Faculty of Science, Beni-Suef University, Beni-Suef, Egypt

**Keywords:** archaea, *Haloferax* sp, cobalt contamination, growth promoting, extreme growth condition, stress

## Abstract

Soil contamination with cobalt (Co) negatively impacts plant growth and production. To combat Co toxicity, plant growth-promoting microorganisms for improving plant growth are effectively applied. To this end, unclassified haloarchaeal species strain NRS_31 (OL912833), belonging to *Haloferax genus*, was isolated, identified for the first time, and applied to mitigate the Co phytotoxic effects on maize plants. This study found that high Co levels in soil lead to Co accumulation in maize leaves. Co accumulation in the leaves inhibited maize growth and photosynthetic efficiency, inducing oxidative damage in the tissue. Interestingly, pre-inoculation with haloarchaeal species significantly reduced Co uptake and mitigated the Co toxicity. Induced photosynthesis improved sugar metabolism, allocating more carbon to defend against Co stress. Concomitantly, the biosynthetic key enzymes involved in sucrose (sucrose-P-synthase and invertases) and proline (pyrroline-5- carboxylate synthetase (P5CS), pyrroline-5-carboxylate reductase (P5CR)) biosynthesis significantly increased to maintain plant osmotic potential. In addition to their osmoregulation potential, soluble sugars and proline can contribute to maintaining ROS hemostasis. Maize leaves managed their oxidative homeostasis by increasing the production of antioxidant metabolites (such as phenolics and tocopherols) and increasing the activity of ROS-scavenging enzymes (such as POX, CAT, SOD, and enzymes involved in the AsA/GSH cycle). Inside the plant tissue, to overcome heavy Co toxicity, maize plants increased the synthesis of heavy metal-binding ligands (metallothionein, phytochelatins) and the metal detoxifying enzymes (glutathione S transferase). Overall, the improved ROS homeostasis, osmoregulation, and Co detoxification systems were the basis underlying Co oxidative stress, mitigating haloarchaeal treatment's impact.

## Introduction

Microorganisms (e.g., fungi, bacteria, and archaea) have been known for having the capacity to boost plant growth and yield (AbdElgawad et al., 2020). They colonize plant tissues, and this assemblage is referred to as the plant microbiome or the phytomicrobiome. Because plants with these growth-promoting microbiomes can produce and grow more, there is an increasing interest in employing them to manage plant growth under various stress conditions (Booth et al., 2002; Subramanian et al., 2016).

Among different microorganisms, archaea are a vital component of the plant microbiome because they interact with plants and protect them from biotic and abiotic stresses (Moissl-Eichinger et al., 2018). Archaea exist ubiquitously, may be present freely or in association with plants, and occur as ecological niches of high pH, temperature, and salinity. Unfortunately, most microbiome studies focused on dominating bacteria and fungi rather than archaea. However, the evidence that archaea are the vital components of plant microbiomes has stimulated scientific interest in plant-associated archaea (Taffner et al., 2019). In various plant species, including scots pine, maize, rice, and many aquatic plant species, archaea represent a stable microbiome component (Hardoim et al., 2015). Archaea are typically present in the plant rhizosphere, with fewer numbers in the endosphere and phyllosphere, because rhizodeposits (e.g., exudates, border cells, mucilage) constitute the fundamental driving force in the regulation of microbial diversity. Furthermore, some species of archaea can survive in both aerobic and anaerobic environments, and this advantage allows archaea to flourish in the plant rhizosphere as it generates both aerobic and anaerobic zones (Trivedi et al., 2019). Among their different roles, archaea appear to have the potential to promote plant growth, improve nutrient supply, boost the defense system, and protect plants against various abiotic stresses (Im et al., 2009; Taffner et al., 2018; Alori et al., 2020; Jung et al., 2020). Recently, few studies highlighted potential interactions between archaea and plants, such as the production of indole acetic acid and siderophores, sulfur cycling (Leigh, 2000); phosphorus-solubilization (Yadav et al., 2015), enhancing plant stress responses, ammonia oxidation, and nitrogen-fixing methanogens (Leigh, 2000), which might be attributed to plant growth-promoting effects. Thus, it could be expected that archaea might interact with terrestrial plants through soil nutrient cycling. However, archaea are an understudied group of microorganisms, and our knowledge of terrestrial archaea and their interactions with plants is limited. Notably, the benefits of plant–archaea relationship may be a promising, reliable, and sustainable strategy, as they have plant growth-promoting attributes such as IAA and siderophore production, phosphorus solubilization, nitrogen fixation, sulfur cycling, dissimilatory nitrate reduction, and ammonia oxidation (Yadav et al., 2017; MacLeod et al., 2019). They also improve nutrient supply and ameliorate the adverse effects of multiple stresses, which contributes to increasing crop production (Alori et al., 2020).

Maize is one of the most important crops worldwide, third to only wheat and rice (Akongwubel et al., 2012). Maize suffers heavily from metals in the soil and other environmental stress conditions (Abdoulaye et al., 2019; AbdElgawad et al., 2020; Gao et al., 2020), which can severely inhibit its growth and productivity. Among heavy metals, Cobalt (Co) might cause severe damage to plant cells at excess concentration, resulting in reduced growth and biomass. In different plants, including maize, Co toxic effects might be attributed to DNA damage, interactions with DNA repair systems, DNA–protein cross-links, and sister chromatid exchange directly (Erturk et al., 2013). Moreover, like other heavy metals, Co generates reactive oxygen species (ROS), which cause oxidative damage, damage to biomolecules and membranes, reflected as lipid peroxidation, and trigger protein carbonylation (Pandey and Sharma, 2002; Pandey et al., 2009). Maize, like other plants, has an antioxidant defense system to cope with the lethal effects of ROS (Rellán-Álvarez et al., 2006; Rizvi and Khan, 2018), but unfortunately, adverse growth conditions lead to decreased growth, productivity, and survival, resulting in poor yields or crop failure (ten Berge et al., 2019). To increase maize production, we suggest modifying microbiomes, which will aid in the enhancement of nutrient uptake and yield, control pests, and mitigate plant stress responses.

Due to the beneficial impacts of archaea, we hypothesized that supplementation of soil with archaea would trigger the growth, neutralize the toxic effects of heavy metals, and boost the defense system in maize. To our knowledge, there are no published studies on the impacts of archaea on maize plants under heavy metal stress such as Co and its ability to squash the toxic effects of Co. The primary objective was to evaluate the effect on the growth and photosynthetic rates of maize in the presence and absence of metal stress. Furthermore, we aimed to evaluate the effect of growth-promoting archaea on osmoregulation, oxidative stress, and antioxidant defense system of maize under heavy metal stress.

## Materials and Methods

### Sampling, Isolation, and Growth Conditions of Haloarchaea Strains

In April 2019, sediment and brine samples were collected from a solar saltern from the coast of Jeddah, Saudi Arabia (21°10'16.04”N, 39°11'5.94”E) into the 1,000-ml clean Pyrex bottles. About 500 ml of brine samples was taken from the border and the center of the hypersaline location at a depth of 5–10 cm in sterile glass bottles. Similarly, approximately 50 g of sediments was scraped and placed in polyethylene bags using a sterile spatula at a depth of 0–5 cm from the surface. Both the brine and sediment samples were transported in an ice box upon arrival to the laboratory. At each sampling point, triplicate sampling (*n* = 3) was performed. All samples were kept at 4°C and subjected to a microbiological examination within 24 h of their arrival.

Isolation of haloarchaea from sediment and brine was done according to the study of Dyall-Smith et al. (2011), which involves that different halophilic media, Eimhjellen medium (EM) (Catherine et al., 2001), HE medium (Torreblanca et al., 1994; Catherine et al., 2001), and DSM 97 (DasSarma et al., 2001), with NaCl concentrations ranging from 10 to 25% (w/v), were used. Pure isolates were obtained by successive cultivation on DSM 97 and also maintained on the same medium.

### Morphological and Biochemical Characterization of the Isolated Haloarchaea

A total of five distinct isolates grown on 10% (w/v) NaCl concentration were selected for further investigation, which are designated as NRS_30, NRS_31, NRS_32, NRS_33, and NRS_34. For the identification of the isolated haloarchaeal strain, morphological and biochemical screenings were carried out according to the proposed minimum standards for the description of new taxa in order Halobacteriales (Oren et al., 1997). Organic acids' production in the culture media was estimated using high-performance liquid chromatography (HPLC) (Shimadzu HPLC system, SCL-10 AVP) coupled with a SUPELCOGELC-610H,30 cm ×7.8 mm ID column and UV detector (LaChrom L-7455 diode series). The mobile phase used was phosphoric acid (0.1%, v/v), which run at a flow rate of 0.45 ml/min^−1^; column temperature: 30 °C; flow rate: 0.5 ml/min; Det.: UV 210 nm. The coefficient of determination of the standard calibration curves was 0.993 ≤ r2 ≤ 0.999 and the limit of detection ranged from 0.05 to 9.40 mg/kg. The organic acids' recovery ranged from 89.5 to 98.9% for target organic acids. Phosphate solubilization was tested by spot inoculation of 10 μl of archaeal suspension to media which contains hydroxyapatite. After incubation for 10 days, the culture suspension was centrifuged (11,000 g; 10 min), and the soluble P content in the supernatant was spectrophotometrically estimated by ascorbic acid method (Murphy and Riley, 1962). Determination of indole acetic acid (IAA) and siderophore production was done as already reported (Gordon and Weber, 1951; Schwyn and Neilands, 1987), respectively, to investigate growth-promoting capacity of haloarchaeal isolates. All methods used for physiological and biochemical characteristics were modified by adding 10% (w/v) NaCl to the growth media.

### Growth on Different NaCl Concentrations

The selected isolates, obtained after 2 weeks of incubation at 37°C, were tested for the growth in NaCl concentration ranging from 0.0 to 10% according to the method described by Oren et al. (1997). About 1 ml of aliquot cell culture was measured at 600 nm on UV-visible spectrophotometer (UV-2600 Series, SHIMADZU) at the intervals of 48 h, and the non-inoculated medium was considered as a control.

### Molecular Identification of the Potential Isolate

The genomic DNA of the promising isolate was extracted using a modified approach described by Maloy (1990). The 16S rRNA gene was amplified using a pair of archaea-universal primers (Invitrogen, USA), 5'-ATTCCGGTTGATCCTGCCGG-3' primers (positions 6–25 in *Escherichia coli* numbering), and 5'AGGAGGTGATCCAGCCGCAG-3' primers (positions 1,540–1,521), whereas the polymerase chain reaction (PCR) conditions were adjusted as previously described (Ventosa et al., 2004). To prepare the samples for sending to MacroGen Company in Korea according to their instructions, about 50 ng/L of each PCR product was employed. The sequences of the single PCR product were analyzed using BLAST (http://www.ncbi.nlm.nih.gov/BLAST) to establish a preliminary identification of the strain.

A phylogenetic tree was constructed using the neighbor-joining method (Tamura and Nei, 1993). The optimal tree with the sum of branch length = 45.13652293 is shown. The percentage of replicate trees in which the associated taxa clustered together in the bootstrap test (500 replicates) are shown next to the branches (Kumar et al., 2018). The tree is drawn to scale, with branch lengths in the same units as those of the evolutionary distances used to infer the phylogenetic tree. The evolutionary distances were computed using the maximum composite likelihood method (Felsenstein, 1985) and are in the unit of the number of base substitutions per site. This analysis involved 10 nucleotide sequences. All ambiguous positions were removed for each sequence pair (pairwise deletion option). There were a total of 2,910 positions in the final dataset. Phylogenetic analyses were conducted in MEGA X.

### Experimental Setup, Plant Materials, and Growth Conditions

Seeds of heavy metal stress-tolerant plant variety (*Zea maize* L. cv Giza 119) were used. Maize seeds were surface-sterilized with 70% ethanol and 1% sodium hypochlorite. A total of three seeds were grown in pots (15 × 20 cm) containing artificial soil. The artificial soil composition was mainly 68% of sand, 13.4% of silt, 7.2% of kaolinite clay, and 8.7% of sphagnum peat. It also contains organic matter (5.2%) with cation exchange capacity of 8.8 cmol kg^−1^. The haloarchaea strains (NRS_31 (OL912833) was freshly grown in 100 ml Eimhjellen medium (EM) and incubated for 72 h at 27°C with shaking at 200 rpm in shaker. Cell density was determined by measuring absorbance of cultures at A600 nm. At A600 nm = 1, cells were centrifuged at 10,000 × g for 10 min, and the cells were washed in saline solution (12%) and resuspended in 10 ml of archaea-free culture medium. Before starting the experiment, the soil was sterilized to remove undesirable and pathogenic microorganisms. The soils were divided into 2 groups: the first group was incubated with unclassified haloarchaeal species strain NRS_31 (OL912833) (10 ml per 0.5 kg/soil), and the second group was incubated with archaea-free culture medium (10 ml per 0.5 kg/soil). After 3 days of inoculation, Co dose (200 mg/kg soil) was applied using CoSO_4_ as Co source. The dose was selected after preliminary experiments on the impact of various concentrations of Co (5–400 mg/kg soil) on maize growth. Moreover, the lowest salinity concentration NaCl (0.25%), at which the archaea species could effectively grow, was applied to the soil. According to our preliminary experiment, the 0.05% did not significantly affect the maize growth under control and Co stress conditions. Irrigation of treated and non-treated pots was done daily up to 60% (soil water content). Plants were maintained in a controlled greenhouse at 21/18°C,16-/8-h day/night, and 60% humidity; they were regularly irrigated. After 4 weeks of sowing, shoot system of the three plants in each pot was harvested as a biological replicate, and 2 pots (12 plants) were harvested per each treatment. The fresh and dry weights of maize shoot were determined, and fresh samples were kept at −80°C for biochemical analyses.

### Soil Analysis

Heavy metals were determined by mass spectrometry (ICP-MS, Finnigan Element XR, Scientific, Bremen, Germany), whereas Co and soil were extracted in HNO_3_/H_2_O (5:1). A digital pH meter (3000 CE) and a conductivity meter (Jenway 3305) were used to measure the pH and EC of the soil, respectively, in a soil–water extract (1:5 w/v). Using a spectrophotometer (Shimadzu UV–Vis 1601 PC, Japan), phenolic acids measured set to 700 nm. About 20 g of soil was mixed with 100 ml of distilled water in a flask and shaken for 12 h. The phenolic content of the soil extract was then determined after filtration. Using gallic acid as a standard, the phenolic content of the soil was measured according to Zhang et al. (2006). According to Jackson (1962), the proportion of CaCO_3_ was calculated. According to Jones (2001), the organic matter (OM) was calculated. Mineral nutrients were extracted in a 5:1 solution of HNO_3_/H_2_O and analyzed using mass spectrometry ICP-MS, PerkinElmer SCIEX, ELAN DRC-e, Scientific, Bremen, Germany). A standard mixture of multi-element calibration standard 3 and no Hg was prepared in 1% nitric acid.

### Photosynthesis

A portable photosynthesis device was used to determine the light saturated photosynthetic rate (LI-6400; LI-COR). In the leaf chamber, the CO_2_ concentration was fixed at 400 μmol mol^−1^, whereas the temperature was determined at 25°C, and Photosynthetic Photon Flux Density (PPFD) was 1,100–1,200 μmol m^−2^ s^−1^, provided by a 6400-02 LED.

### Quantification of Oxidative Damage Markers

Following FOX1 method, the concentration of H_2_O_2_ was estimated by monitoring the Fe^3+−^xylenol orange complex at 595 nm as indicated by peroxide-mediated oxidation of Fe^2+^, followed by reaction of Fe^3+^ with xylenol orange (Jiang et al., 1992). Determination of lipid peroxidation was done using thiobarbituric acid–malondialdehyde (TBA-MDA) reagent (Hodges et al., 1999). The absorbance was detected at 440, 532, and 600 nm, and the content was represented as nmol g^−1^ FW. Protein carbonyls were detected as oxidative damage markers using Cayman Chemical's Protein Carbonyl Colorimetric Assay Kit (Ann Arbor, MI) as described by Levine et al. (1994).

### Quantification of Antioxidant Parameters

In 80% ethanol, the total antioxidant capacity (FRAP) and antioxidants (phenolics and flavonoids) were extracted. Centrifugation was done at 14,000 g, 4°C, for 25 min, and then, the FRAP test (TPTZ [0.01 mM] in HCl [0.04 mM], acetate buffer [0.3 M, pH3.6], and FeCl_3_.6H_2_O [0.02 M]) was performed using Trolox (0 to 650 M) as already described by Benzie and Strain (1996). Folin-Ciocalteu test was used to assess the content of the polyphenols in sample extractions (Demirbaş and Acar, 2008). The modified aluminum chloride method was used to calculate the flavonoid content. HPLC analysis was used to assess ascorbate (ASC) and glutathione (GSH) (Shimadzu, Hertogenbosch, the Netherlands). Metabolites were separated on the reversed-phase HPLC column (Polaris C18-A [100 mm x 4.6 mm], particle size [3 μm], and 42°C). The diode array detector (DAD) was used to detect ASC and GSH (Zinta et al., 2014). The elution buffer was as follows: 2 mM KCl, pH 2.5 adjusted with O-phosphoric acid. Meta-phosphoric acid (6 %, w/v) was used for extraction of plant samples (Zinta et al., 2014). Samples were diluted 1:4 in mobile phase (2 mm KCl, pH set at 2.5 with H_3_PO_4_) prior to injection. Total ASC (ASC plus DHA) was determined by reducing with dithiothreitol in Tris solution (pH of 6). After 1-h incubation at room temperature, samples were acidified again by the addition of 300-μl mobile phase and kept at 4°C until injection. The DHA concentration was estimated as the difference between the reduced and total ASC concentration. Chromatogram analysis was performed with the Class VP software package (ClassVP 5.0, Shimadzu).

For antioxidant enzyme activity, peroxidase (POX) and superoxide dismutase (SOD) proteins were extracted using K-phosphate extraction buffer (50 mM and pH 7.0) containing polyvinylpolypyrrolidone (PVPP) (10%, w/v), Triton X-100 (0.25%, v/v), and phenylmethylsulfonyl fluoride (PMSF) (1 mM). POX and SOD activities were measured by the oxidation of pyrogallol at 430 nm (Kumar and Khan, 1982) and the inhibition of NBT reduction at 560 nm (Dhindsa et al., 1981), respectively. Dehydr-ASC reductase (DHAR), GSH reductase (GR), ascorbate peroxidase (APX), and monodehydro-ASC reductase (MDHAR) were extracted in extracted in 50 mM MES/KOH buffer (pH 6.0), containing 2 mM CaCl_2_, 40 mM KCl, and 1 mM ascorbic acid, and evaluated spectrophotometrically, based on the protocols described in Murshed et al. (2008) using MES/KOH (0.05 M). The activity of catalase (CAT) was extracted in a 50 mM potassium phosphate (pH 7.0) containing 0.4 mM ethylenediaminetetraacetic acid (EDTA), 0.2 mM PMSF, 2% (w/v) insoluble PVPP, and 1 mM ascorbic acid, and it was measured by monitoring the rate of decomposition of H_2_O_2_ at 240 nm (Aebi, 1984). The glutathione peroxidase (GPX) was extracted in a 50 mM potassium phosphate (pH 7.0) containing 0.4 mM EDTA, 0.2 mM PMSF, and 2% (w/v), and its activity was assayed by following the reduction of nicotinamide adenine dinucleotide phosphate (NADPH) at 340 nm (Drotar et al., 1985). The total soluble protein concentration was measured by Lowry technique (Lowry et al., 1951).

### Osmo-Protectant

For extraction of sugars, Tris-acetate-EDTA (TAE) buffer (50 mM, pH 7.5) containing 0.02% Na-azide, 10 mM mannitol, 0.15% Polyclar, 12 mM NaHSO_3_, 1 mM mercapto-ethanol, and 2 mM PMSF were used. Centrifugation of the extracts was done at 15,000 g and 4°C, for 10 min. A part of the extract was heated for 5 min at 90°C. After cooling and centrifugation at 14,000 g (4°C and 5 min), the supernatants were transferred to a mixed bed Dowex column of 300 μl Dowex H^+^, 300 μl Dowex Ac^−^, and both 100–200 mesh. After elution with ddH_2_O, the concentrations of different sugars, i.e., glucose, fructose, sucrose, and raffinose were measured (HPAEC-PAD) (AbdElgawad et al., 2014). Analysis and detection were performed at 32°C and the flow rate was 200 μl per min. A 20 μl of sample was injected on a Guard CarboPac PA 100 (2 × 50 mm) in series with an analytical CarboPac PA 100 (2 × 250 mm). Sugars were eluted in 90 mM NaOH, with an increasing sodium acetate gradient: from 0 to 6 min, the sodium acetate concentration increased linearly from 0 to 10 mm; from 6 to 16 min, from 10 to 100 mm; from 16 to 26 min, from 100 to 175 mm. The columns were then regenerated with 500 mM sodium acetate for 1 min and equilibrated with 90 mM NaOH for 9 min for the next run. Data were recorded and processed with Chromeleon software.

The non-heated supernatant was used for measuring the activity of the key sugar enzymes. The activity of invertases enzyme was measured in TAE buffer with 100 mM sucrose. Then, reaction mixtures were incubated at 30°C, and the reactions were stopped by heating an aliquot for 5 min at 90°C in a water bath. Similarly, by following the above steps, glucose and fructose concentrations were detected.

The activity of sucrose phosphate synthase (SPS) was measured in 1 ml of (4-(2-hydroxyethyl)-1-piperazineethanesulfonic acid) HEPES buffer at pH 8 contained UDP-glucose and mM fructose-6-phosphate at a temperature of 37°C for 20 min, and then, the reaction was stopped by adding NaOH (30%). The reaction mixture containing citrate and glycogen initiated the starch synthase (Nishi et al., 2001).

Amino acids (proline) were extracted in 2 ml of 80% ethanol and spiked with norvaline as an internal standard, and then, centrifugation was performed at 14,500 rpm for 18 min. The supernatant was then evaporated, and the pellets were resuspended in 1 ml chloroform. Simultaneously, 1 ml HPLC-grade water was used for re-extraction of the residue. After centrifugation at 14,000 rpm for 20 min, the supernatant was mixed with the pellets suspended in chloroform. Such combined extract was subjected to centrifugation for 10 min at 14,000 rpm. Afterward, filtration of the aqueous phase was performed through Millipore micro-filters (0.2 μm pore size) before assaying amino acid levels. Proline was determined by using a Waters Acquity UPLC-TQD system equipped with a BEH amide 2.1 X 50 column (Sinha et al., 2013). About 10 μl of each sample was injected and eluted with a gradient of solvent A (0.1% formic acid in H_2_O) and solvent B (0.1% FA in acetonitrile) over 7.5 min at 0.5 ml/min. Flow rate was set at 0.3 ml/min^−1^, the column temperature was maintained at 30°C, and the sample temperature was set at 20°C.

In the same context, the protocols outlined by AbdElgawad et al. (2015) were used for the determination of enzyme activities which are incorporated in proline metabolism. Plant tissue was extracted (100 mg ml^−1^ 50 mM Tris-HCl, pH 7.4, 2% (w/v) polyvinylpyrrolidone, 4 mM DTT, 10 mM MgC12, 1 mM EDTA, 10% glycerol, and 2 mM PMSF). Pyrroline-5-carboxylate synthetase (P5CS), pyrroline-5-carboxylate reductase (P5CR), pyrroline-5-carboxylate dehydrogenase (P5CDH), and proline de-hydrogenase (ProDH) were extracted in 100 mg ml^−1^ 50 mM Tris-HCl, pH 7.4, 2% (w/v) polyvinylpyrrolidone, 4 mM DTT, 10 mM MgC1_2_, 1 mM EDTA, 10% glycerol, and 2 mM PMSF. The activities of all enzymes (P5CS, P5CDH, and PRODH) were measured by monitoring the reduction of NADH at A340 (Shabbaj et al., 2021), the P5C-dependent NADH oxidation at A340 (Lutts et al., 1999), and production γ-glutamyl hydroxamate at A535. P5CS was assayed in 50 mM tris-HCl pH 7.0, and Prod was assayed in Tris-HCl buffer (200 mM, pH 8.0). Using Bovine Serum Albumin (BSA) as a standard, the protein concentrations were measured as assessed by Lowry et al. (1951).

### Statistical Analysis

All results were expressed as the mean values of five biological replicates (*n* = 5). Statistical analysis was performed using one-way ANOVA in the SPSS 22 (Tukey's test, *p* ≤ 0.05). Data normality was checked using Levene's test. Meanwhile, the hierarchical cluster analysis Euclidean distance was performed using the R stat software package (version 4.5.0, the R).

## Results

### Biochemical Characterization of the Isolated Haloarchaea

A total of five bacterial strains (a transparent (NRS_31) and shades of red color (NRS_30, 32, 33, and 34) were isolated. The characterization was based on the biochemical traits, i.e., organic acid production, phosphate solubilization, siderophore, and IAA production (Table 1). In this study, almost all bacterial strains were capable of producing organic acids (citric, gluconic, formic, fumaric, propionic, succinic, and tartaric acids). However, strains NRS_33 and NRS_34 and NRS_30 and NRS_32 were not able to produce gluconic acid and formic acid, respectively. Meanwhile, succinic and tartaric acids were not formed by NRS_34 and NRS_32, respectively. Interestingly, strain NRS_31 had the highest production of organic acids compared to the other studied haloarchaea (Table 1).

**Table 1:** The ability of the selected isolate archaea strains to produce organic acid, siderophore, and auxin (IAA) compounds and to solubilize phosphate.

	**NRS_30**	**NRS_31**	**NRS_32**	**NRS_33**	**NRS_34**
**Organic acid (μg/mL)**					
Citric acid	26.8 ± 1	44.1 ± 0.3	1.9 ± 0	18.5 ± 0.8	16 ± 0.2
Gluconic acid	25.6 ± 1.5	35.1 ± 0.3	2 ± 0.1	ND	ND
Formic acid	ND	37.8 ± 1	ND	17.6 ± 1.1	13.8 ± 0.4
Fumaric acid	29.9 ± 1	38.5 ± 0.2	2.1 ± 0	20.9 ± 0.6	8.9 ± 0.1
Propionic acid	30.8 ± 1	40.1 ± 1.2	2.2 ± 0.1	19.2 ± 0.5	15.7 ± 0.9
Succinic acid	2.9 ± 0.2	19.1 ± 0.3	2.1 ± 0.1	21.4 ± 0.7	ND
Tartaric acid	29.3 ± 2	35.1 ± 1.4	ND	19.6 ± 1.1	15.3 ± 0.4
Phosphate solubilisation (mg/L)	8.8 ± 0.7	40.1 ± 1.7	2.1 ± 0	18.9 ± 0.9	14.6 ± 0.7
Siderophore (μg/g)	1.8 ± 0.1	2.6 ± 0.1	0 ± 0	0.1 ± 0	0 ± 0
IAA (μmol/g)	ND	0.59 ± 0	0.02 ± 0	0.06 ± 0	ND

*The values were expressed as the mean of 3 replicates (mean ± S.D). ND, Not detected*.

On the other hand, the potentiality of haloarchaea as phosphate solubilizers is one the important traits for the selection of bacteria. Phosphate solubilization by haloarchaea mostly depends on the availability of different phosphate sources. Our results showed that strain NRS_31 had the highest ability of phosphate solubilization (45.6 mg/L), whereas the other haloarchaea had a lower phosphate solubilization activity (Table 1).

The production of growth regulators, such as IAA, is among the direct effects of plant growth-promoting bacteria, and thus, we investigated the ability of bacterial strains to synthesize IAA. Only strains NRS_31 and NRS_33 had the capability of IAA synthesis (0.6 μmol/g and 0.1 μmol/g, respectively), and thus, NRS_31 had the highest production of IAA among the other investigated haloarchaea (Table 1). Moreover, the production of siderophore is known to protect the plant against pathogens. Among the tested haloarchaea, NRS_30, NRS_31, and NRS_33 were able to produce siderophores (1.8 μg/g, 2.6 μg/g, and 0.1 μg/g, respectively) (Table 1). Moreover, out of the five tested isolates, only strain NRS_31 exhibited a distinct range of growth in the presence of low NaCl concentrations (0.4 to 1.7 M) (Table 2). Based on the above results, strain NRS_31 was selected as the most active archaea and was further identified by molecular characterization.

**Table 2:** Soil structure, physical and chemical composition under Co (Co) and/or archaea treatment.

	**Ctr**	**Co**	**Archaea**	**Archaea-Co**
Sand%	60.0 ± 0.8a	58.7 ± 0.5a	57.4 ± 0.5a	56.1 ± 0.4a
Silt%	24.4 ± 0.3a	23.9 ± 0.2a	23.3 ± 0.2a	22.8 ± 0.2a
Clay%	8.04 ± 0.1a	7.85 ± 0.1a	7.67 ± 0.1a	7.5 ± 0.1a
E.C. ds/m	1.66 ± 0.1a	1.63 ± 0a	1.59 ± 0a	1.6 ± 0a
pH	6.9 ± 0a	6.73 ± 0a	6.57 ± 0.1a	6.4 ± 0a
CaCO3 g/Kg	31.3 ± 1a	28.6 ± 1a	38.1 ± 0.6b	35.8 ± 3b
Organic matter (g/Kg)	0.96 ± 0.1ab	0.98 ± 0ab	1.45 ± 0a	1.2 ± 0.1a
**Macro nutrient mg/Kg**				
Total N mg/Kg soil	158 ± 0.9a	144 ± 1.2ab	160 ± 1.3a	157 ± 1a
NH4-N	6.53 ± 0.4a	6.44 ± 0.1a	6.3 ± 0.1a	6.2 ± 0.1a
NO3-N	108 ± 0.6a	105 ± 0.8a	103.1 ± 0.9a	100 ± 0.7a
Na g/kg	0.45 ± 0a	0.45 ± 0a	0.44 ± 0a	0.4 ± 0a
Total P mg/Kg soil	128 ± 3.3b	126 ± 12b	155 ± 2.7	154 ± 1aa
K mg/Kg soil	110 ± 5.4ab	112 ± 4.8ab	124 ± 2.3a	126 ± 10a
Ca	12.0 ± 0.2ab	12.1 ± 0.5ab	14.1 ± 0.3a	14.6 ± 1a
Mg	5.44 ± 0.3b	5.8 ± 0.9b	7.8 ± 0.1a	8 ± 0.5a
Fe	6.57 ± 0.2b	8.2 ± 0.3a	6.7 ± 0.2b	8 ± 0.5a
**Micronutrient mg/Kg**				
S	1.42 ± 0.1ab	1.6 ± 0a	1.47 ± 0ab	1.3 ± 0ab
Mn	1.85 ± 0.1a	1.82 ± 0a	1.78 ± 0a	1.7 ± 0a
Zn	1.46 ± 0.1ab	1.64 ± 0a	1.25 ± 0b	1.49 ± 0ab
Cu	3.37 ± 0.1	3.28 ± 0	3.2 ± 0	3.1 ± 0
Co (mg/Kg)	0.08& ± 0c	144.68 ± 2.3b	0.065 ± 0c	170 ± 0.9a

*The mean values and standard errors were calculated based on 3 biological replicates (p < 0.05; n = 3). The significant changes among the treatments were indicated by the different characters*.

### Molecular Identification of a Potential Strain

**Figure 1:**
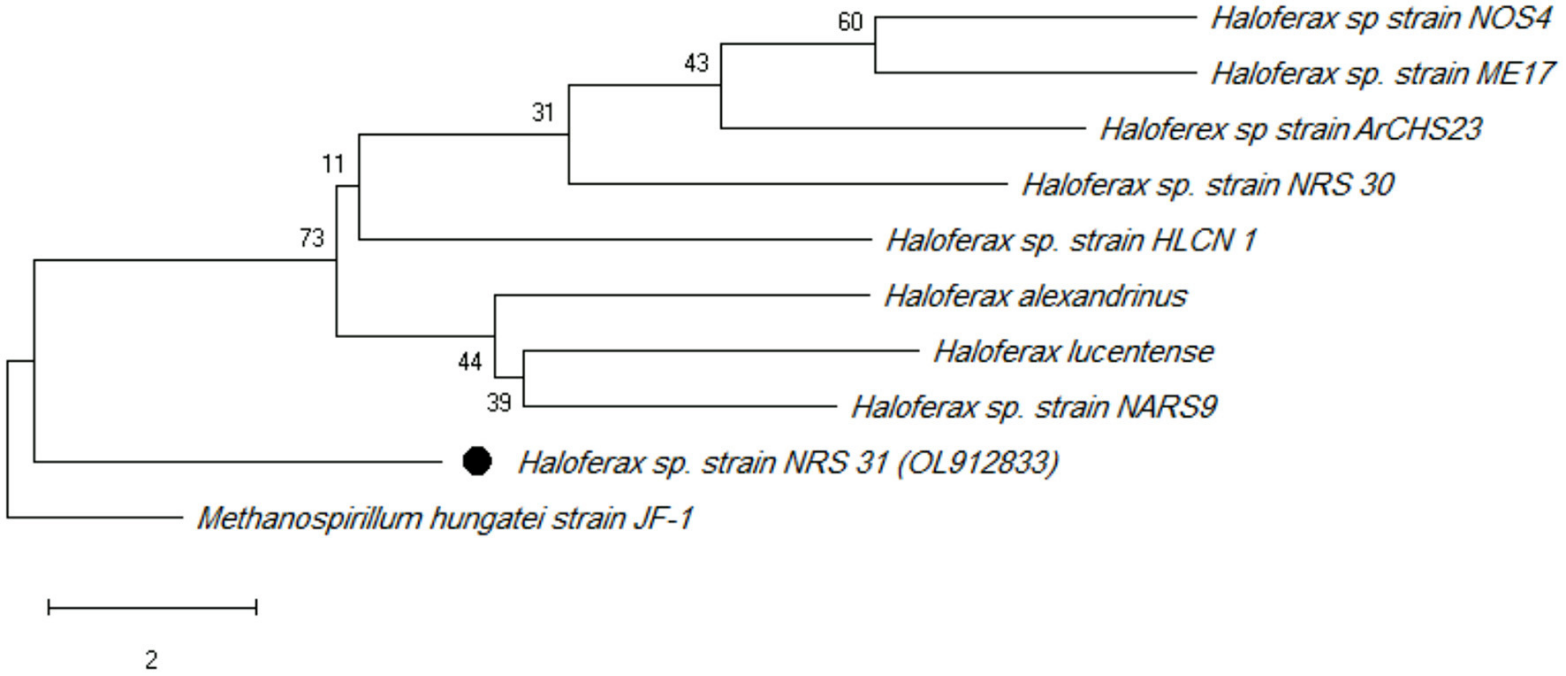
Maximum likelihood phylogenetic tree (using MEGA software) based on 16S rRNA gene sequences showing the relationship between *Haloferax sp* strain NRS_31 and closely related taxa. Scale bar indicates 0.01 substitutions per nucleotide position.

The phylogenetic tree was constructed using type strains. According to several studies such as Zhang et al. (2020), *Methanospirillum hungatei* JF-1T (a methane-producing archaeon) was used as outgroup. With 99% sequence similarity, 16S rRNA gene sequencing revealed that the potential strain NRS_31 is a member of *Haloferax* sp. The haloarchaeal strain's 16S rRNA gene data have been deposited in the NCBI and GenBank nucleotide sequence databases under the accession number OL912833 (Figure 1).

### Effect of Archaea on Soil Structure and Composition

To evaluate the effect of treatment with Co and/or strain NRS_31 on the soil supporting the growth of maize plant in relation to its metabolic contents, we investigated the physical properties (sand%, silt%, clay%, electrical conductivity (EC), pH, CaCO_3_, and organic matter content) and chemical composition (macronutrients, i.e., total N, NH4-N, NO3-N, Na, total P, K, Ca, Mg, and Fe, and micronutrients, i.e., S, Mn, Zn, Cu, and Co) of soil (Table 2). Regarding the physical properties of soil, the individual and combined treatments with Co and/or archaea did not induce significant changes in the percentages of soil sand, silt, and clay, as compared to the control samples. The EC and pH were not affected by any of the treatments, when compared to the control. Moreover, the CaCO_3_ and organic matter contents of soil were enhanced, only when plants were exposed to treatment with strain NRS31, or when it was combined with Co stress (by about 10%), as compared to the control samples, while the organic matter content was not affected by any of the treatments.

The chemical composition of the soil revealed that treatment with Co only enriched Co content of the soil by 360%, but it did not affect most of the measured nutrients, as compared to the control. Meanwhile, only the Mg content was increased by 40% in NRS_3-treated maize plants. Moreover, no changes were observed in the soil contents of total N, NH4-N, NO_3_-N, and Na in plants exposed to the combined treatment of Co and strain NRS_31, whereas the Mg, Fe, and Co contents were increased (by 60, 30, and 360%, respectively) as compared to the control plants. Thus, it could be observed that the combination of Co and strain NRS_31 had a more pronounced effect on physical and chemical characteristics of the soil.

### Effect of Archaea on Growth of Maize

**Figure 2:**
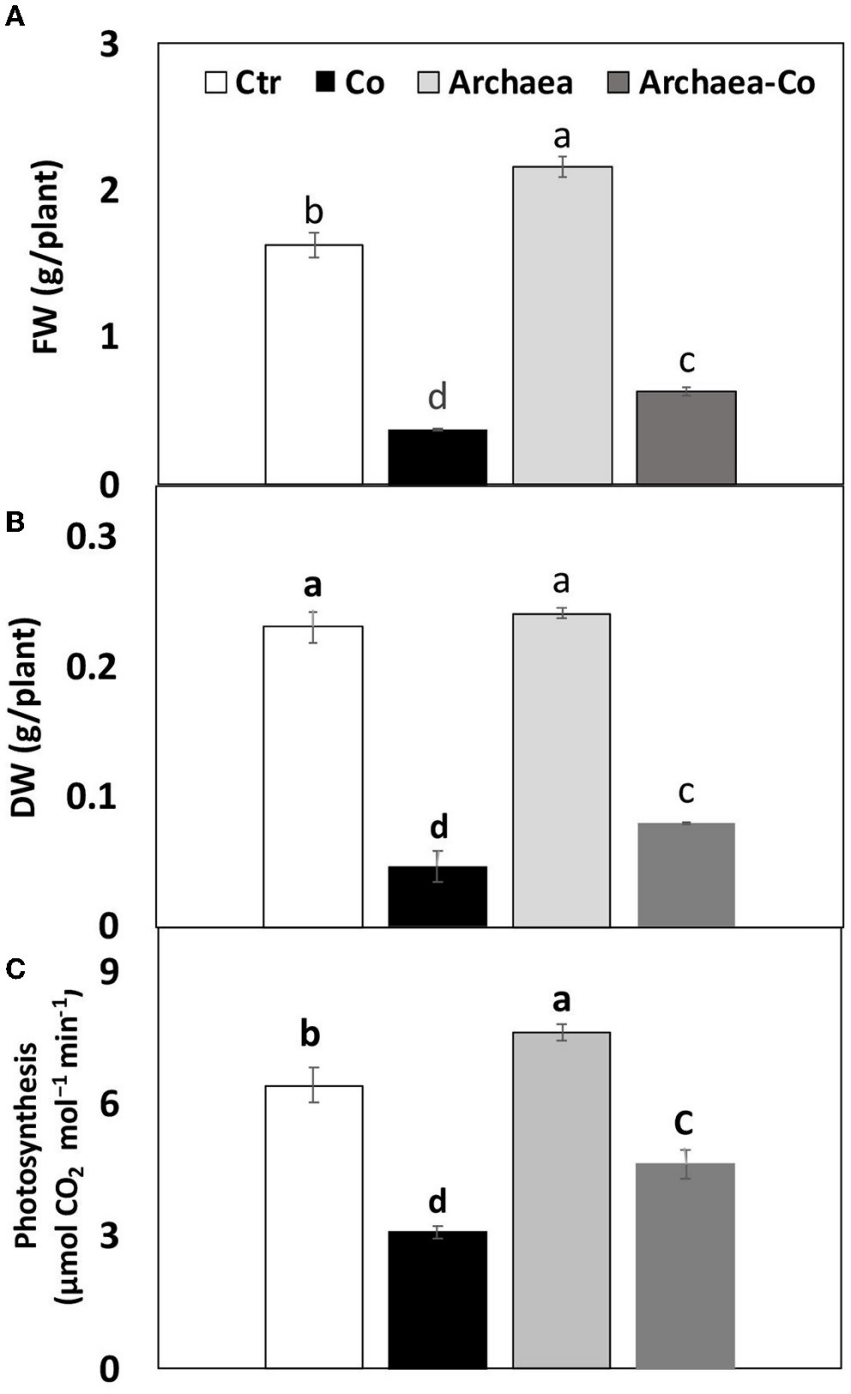
Impact of cobalt (Co) on biomass production **(A)** FW, **(B)** DW, and **(C)** Photosynthesis, expressed as (g/plant), maize inoculated and non-inoculated with strain NRS_31. The mean values and standard errors were calculated based on 3 biological replicates (*p* < 0.05; *n* = 3). The significant changes among the treatments were indicated by the different characters.

Archaea have been known to induce positive effects on plant growth through production of phytohormones, such as IAA, which induce cell differentiation, whereas Co might inhibit vegetative growth. This study has shown a severe reduction in fresh and dry weights of maize plants grown in soil contaminated with Co (300%), as well as under the combined effect of (Co+ Arc) (200%), when compared to the control plants (Figure 2). This reduction in biomass could indicate the inhibition of plant growth by Co. The treatment with strain NRS_31 has partially restored the fresh weight (with a highly remarkable increase by 80%) of the tested plant species, as compared to the control plants, whereas the dry weight was almost not affected. So, the biomass production of maize plants could be enhanced by treatment with strain NRS_31, which might mitigate the harmful effects of Co on plant growth.

### Effect of Archaea on Photosynthesis

Photosynthesis also could be improved by archaea treatment through increased mineral availability and production of growth regulators, such as IAA, whereas Co may cause inhibition of photosynthetic parameters. To understand the induced effects by archaea and/or Co stress on maize plants, we measured the photosynthetic activity under these treatments (Figure 2). Similarly, the decreased biomass accumulation under Co stress was accompanied with a comparable reduction in photosynthetic activity in maize plants grown under Co stress (by 50%), as well as under the combined effect of Co and NRS_31 by 30%, as compared to the control plants. Meanwhile, the photosynthetic rate of maize plants was not changed by treatment with archaea. Overall, the treatment of plants with strain NRS_31 could alleviate the detrimental effect imposed by Co on photosynthetic efficiency.

### Effect of Archaea on Osmoregulation

Bacterial treatment could induce higher levels of sucrose and proline, as a result of the enhanced photosynthetic activity, and thus, bacteria have an indirect effect on osmoregulation. Similarly, Co as a heavy metal could affect the proline and sugar contents of plants. Therefore, to understand the basis for osmoregulation under treatment with Co and/or archaea, we analyzed the changes in sucrose and its metabolizing enzymes (Table 3). The treatment with strain NRS_31 induced significant increases in the soluble sugars and sucrose (by 80 and 100% respectively), whereas Co stress increased the levels of sucrose (by 100%), but did not change the soluble sugars, as compared to the control values. More interesting, a combination of archaea and Co led to more increase in soluble sugars and sucrose by 110 and 200%, respectively, compared with the control plants.

**Table 3:** Effect of cobalt (Co) on sugars and proline levels and their metabolism in maize plants inoculated and non-inoculated with strain NRS_31.

	**Ctr**	**Co**	**Archaea**	**Archaea-CO**
Soluble sugars	4.01 ± 0.23c	4.12 ± 0.26c	7.63 ± 0.19b	9.4 ± 0.36a
Sucrose	2.65 ± 0.14c	4.53 ± 0.16b	4.39 ± 0.2b	6.63 ± 0.39a
Sucrose P synthase	0.24 ± 0c	0.86 ± 0.01a	0.61 ± 0b	0.86 ± 0.01a
Vacuolar invertase	0.54 ± 0.02c	1.14 ± 0.08b	1.39 ± 0.03b	1.98 ± 0.07a
Cytoclic invertase	1.5 ± 0.2b	1.9 ± 0.03ab	1.62 ± 0.25b	2.66 ± 0.01a
Proline	2.77 ± 0.22c	8.16 ± 0.4a	3.52 ± 0.26c	6.17 ± 0.4b
P5CS	2.05 ± 0.04b	4.65 ± 0.15a	2.16 ± 0.07b	4.81 ± 0.15a
P5CR	0.34 ± 0.03c	0.84 ± 0.04b	0.42 ± 0.04c	1.24 ± 0.06a
PRODH	4.7 ± 0.1b	5.9 ± 0.31a	6.06 ± 0.13a	6.09 ± 0.3a

*The mean values and standard errors were calculated based on 3 biological replicates (p < 0.05; n = 3). The significant changes among the treatments were indicated by the different characters*.

For more understanding of the mechanism behind the changes in sugar metabolism, we investigated the sucrose biosynthetic enzyme, i.e., sucrose phosphate synthase (sucrose-P-synthase, SPS), which is involved in synthesis of sucrose. The activity of SPS was induced by treatment with strain NRS_31, and also under Co stress as compared to the control plants. Meanwhile, when archaea were combined with Co, there were higher increments in SPS (320%) compared with the control plants.

In the same context, we measured the changes in sucrose biodegradation enzymes, i.e., vacuolar invertase and cytosolic invertase, which hydrolyze sucrose into glucose and fructose, under the different treatments. Significant increases in vacuolar invertase could be observed in plants treated individually with strain NRS_31, i.e., 30%, when compared to the control plants, but no significant difference was observed for cytosolic invertase. The treatment with Co also enhanced the activities of vacuolar and cytosolic invertases. The combined treatment of both strain NRS_31 and Co induced higher activities of vacuolar and cytosolic invertases (by 280 and 70%, respectively) as compared to the corresponding control plants.

On the other hand, we also evaluated the proline content as a key indicator in osmoregulation under stress conditions. Under treatment with strain NRS_31, the proline content was almost not affected as compared to control values (Table 2). A significant increment in proline was also observed when plants were exposed to Co treatment. A combination of strain NRS_31 and Co treatment remarkably enhanced the proline content (by 270%) compared with the control plants.

To understand the bases of increased proline content, we investigate its biosynthetic enzymes, i.e., pyrroline-5-carboxylate synthase (P5CS) and pyrroline-5-carboxylate reductase (P5CR). In our investigation, treatment with strain NRS_31 did not induce significant changes in P5CS nor P5CR activities, compared with the control plants. A pronounced increase in P5CS and P5CR could be obtained for maize plants grown in soils enriched with Co. A combination of archaea and Co treatment improved the activities of P5CS and P5CR (180% for both) when compared to the control plants.

Regarding the proline biodegradation enzymes, we evaluated the changes in proline dehydrogenase (PRODH), which catalyzes the first step in proline degradation. According to our results, the PRODH activity was enhanced by the sole treatment with archaea or Co. Moreover, the interactive impact of both archaea and Co also induced significant increases in PRODH activity by 50% in comparison with control plants.

Overall, the increased contents of sucrose and proline, particularly under the combined treatment of strain NRS_31 and Co, were accompanied with increased activities of their biosynthetic and degradation enzymes, which could explain the regulatory mechanism used by plants to cope with Co stress. This also could reflect the role of strain NRS_31 in mitigating the adverse effects of Co on plants.

### Effect of Archaea on Oxidative Stress

Co can induce the plant oxidative stress by generation of reactive oxygen species (ROS). Consequently, the destruction of membrane lipids under Co stress could increase the lipid peroxidation and hence the amount of malondialdehyde (MDA). Meanwhile, archaea are able to mitigate the adverse effects induced by stress conditions. Hydrogen peroxide (H_2_O_2_) is also among the generated ROS, which can cause injury to the plant cell. Protein oxidation can occur as a result of generation of ROS, which lead to the modifications of proteins through the oxidation of amino acid side groups.

**Figure 3:**
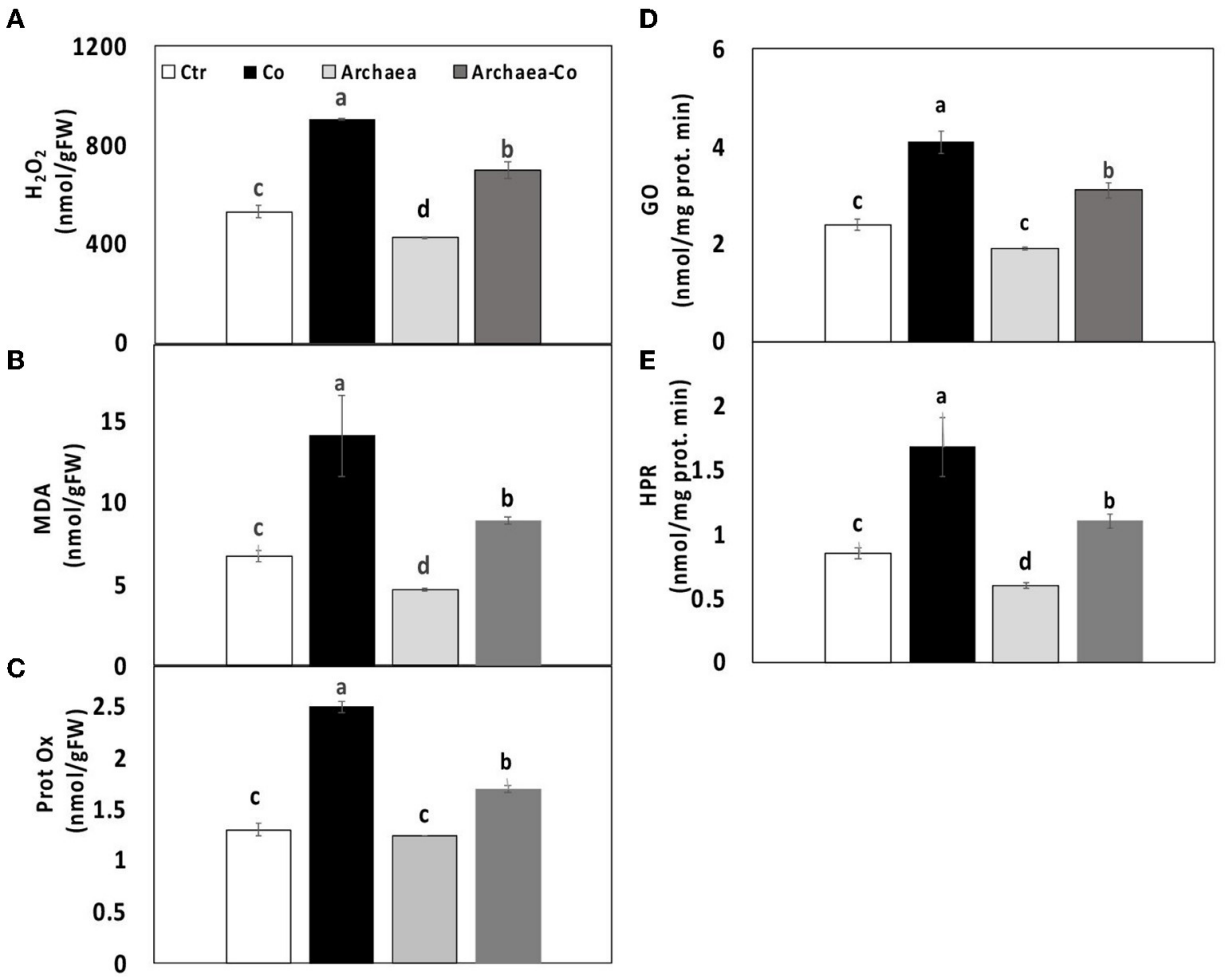
Effect of cobalt (Co) on oxidative status **(A)** H_2_O_2_, **(B)** MDA and **(C)** protein oxidase, **(D)** glycolate oxidase, and **(E)** hydroxypyruvate reductase in maize plants inoculated and non-inoculated with strain NRS_31. The mean values and standard errors were calculated based on 3 biological replicates (*p* < 0.05; *n* = 3). The significant differences among the treatments were indicated by the different characters.

Thus, our results demonstrated the changes in oxidative stress markers of maize plants treated with strain NRS_31, Co, and their combination. The levels of H_2_O_2_ and MDA were reduced due to treatment of plants with strain NRS_31 (by 20 and 30%, respectively), whereas protein oxidation was not changed, as compared to the control plants (Figure 3). However, the amount of H_2_O_2_, MDA, and protein oxidation was greatly enhanced in maize plants after Co treatment, i.e., 80, 120, and 100%, respectively), as compared to their respective control plants, which indicates the responsiveness of plants to the stress conditions. Also, when strain NRS_31 was combined with Co, significant increments were observed in H_2_O_2_, MDA, and protein oxidation (30, 40, and 20%, respectively), as compared to the control plants.

To find an explanation for increasing the levels of ROS, the activities of two important enzymes involved in photorespiration were evaluated. These enzymes are hydroxypyruvate reductase, which is responsible for conversion of hydroxypyruvate into glycerate, and glycolate oxidase, which can oxidize glycolic acid into glyoxylate and also oxidize glyoxylate to oxalate. The activities of both enzymes were enhanced by 100% in maize plants exposed to Co stress. In contrast, the treatment of maize plants with strain NRS_31 reduced the activities of hydroxypyruvate reductase and glycolate oxidase by 30 and 50%, respectively, in comparison with the control plants. When strain NRS_31 was accompanied by Co treatment, the activities of both hydroxypyruvate reductase and glycolate oxidase were increased (30 and 50%, respectively), in comparison with the control plants. Thus, it could be noted that the combined treatment of strain NRS_31 and Co increased the levels of H_2_O_2_, MDA, and protein oxidation, as well as the activities of hydroxypyruvate reductase and glycolate oxidase, which could reflect the role of archaea in mitigating the stress caused by Co treatment.

### Effect of Archaea on Non-enzymatic Antioxidants

To cope with stress conditions, plants might induce antioxidants, which could play a role in mitigating the detrimental effects of heavy metal stress. Archaea also might contribute to increasing the levels of antioxidants to reduce the oxidative stress under Co stress.

In the current investigation, we detected several antioxidants in maize plants under different treatments with Co and/or strain NRS_31 (Table 4). To get an overview about antioxidant defense system, we evaluated the total antioxidant capacity (TAC) using the ferric reducing antioxidant power assay (FRAP), whereas no changes in TAC could be observed in plants exposed to Co stress. The treatment with strain NRS_31 significantly increased the TAC (100%), compared to their control values. The combined treatment with Co and strain NRS_31 caused a more significant increase in TAC (by 130%) when compared to the control plants.

**Table 4:** Effect of cobalt (Co) antioxidant metabolites and enzymes in maize plants inoculated and non-inoculated with strain NRS_31.

	**Ctr**	**Co**	**Archaea**	**Archaea-CO**
Pphenol	3.93 ± 0.08c	6.04 ± 0.3a	4.84 ± 0.1c	7.23 ± 0.1b
Flav	0.17 ± 0c	0.21 ± 0.01c	0.34 ± 0.01b	0.48 ± 0.01a
Toco	31.81 ± 0.6c	43.45 ± 2.5c	106.59 ± 1.6ab	116.54 ± 3.3a
TAC	56.05 ± 1.1c	72.54 ± 4.1c	114.11 ± 1.8b	131.81 ± 4.0a
GSH/TGSH	86.02 ± 1.6b	74.89 ± 4.3b	130.22 ± 2.1a	128.09 ± 4a
GSH	0.04 ± 0c	0.07 ± 0b	0.07 ± 0b	0.09 ± 0a
ASC/TASC	51.0 ± 0.97b	41.61 ± 2.6b	94.0 ± 1.4a	88.8 ± 3.0a
ASC	0.1 ± 0c	0.49 ± 0.01a	0.22 ± 0.01b	0.44 ± 0.01a
POX	3.18 ± 0.06d	5 ± 0.33c	9.7 ± 0.1b	12.81 ± 0.4a
CAT	12.02 ± 0.24d	16.42 ± 0.9c	30.96 ± 0.49b	44.01 ± 1.27a
SOD	187.3 ± 4d	232.4 ± 12c	315.6 ± 5.0b	415.25 ± 12a
APX	1.09 ± 0.0c	4.51 ± 0.0a	3.12 ± 0.05b	4.03 ± 0.12a
DHAR	0.5 ± 0.01c	0.92 ± 0.03b	0.87 ± 0.01bc	1.09 ± 0.03a
MDHAR	0.44 ± 0.01c	0.58 ± 0.03c	0.8 ± 0.01b	1.05 ± 0.03a
GR	3.04 ± 0.06c	3.79 ± 0.2c	5.3 ± 0.08b	8.73 ± 0.3a
GPX	2.86 ± 0.06c	6.55 ± 0.19ab	4.85 ± 0.08b	7.28 ± 0.22a
Glutaredoxins	1.01 ± 0.02c	5.36 ± 0.08b	2.54 ± 0.04b	4.75 ± 0.14a
Thioredoxin	0.08 ± 0c	0.2 ± a	0.15 ± 0b	0.25 ± 0.01a
Pero-redoxins	2.92 ± 0.06d	4.12 ± 0.25c	7.82 ± 0.12b	10.93 ± 0.3a

*The mean values and standard errors were calculated based on 3 biological replicates (p < 0.05; n = 3). The significant differences among the treatments were indicated by the different characters*.

On the other hand, the changes in the individual antioxidants, either soluble antioxidants, i.e., polyphenols, flavonoids, ascorbate (ASC), and glutathione (GSH), or insoluble antioxidants, i.e., tocopherols, were investigated (Table 4). Regarding the soluble antioxidants, there were significant increases in flavonoids, ASC, and GSH levels in maize plants inoculated with NRS_31 strain (170, 300, and 90%, respectively), whereas the polyphenol was not changed as compared to control plants. Co treatment, on the other hand, also significantly increased such measured parameters, but did not affect the flavonoid content. Moreover, a more pronounced increase in polyphenols, flavonoids, ASC, and GSH (120, 320, 300, and 120%, respectively) could be noted in maize plants grown in soils enriched with Co and strain NRS_31, in comparison with the control plants.

In addition, the changes in insoluble antioxidants (tocopherols) were also detected in maize plants under different treatments with strain NRS_31, Co, and their combination, whereas tocopherols were increased in response to the individual treatment with Co. Maize plants treated with strain NRS_31 strain had increased tocopherol content (220%), compared to the control plants. A combination of Co and archaea further induced enhancement in tocopherol levels (240%). Furthermore, we evaluated the ratios GSH/TGSH and ASC/TASC in maize plants subjected to strain NRS_31 and/or Co stress. Co treatment did not induce significant differences in the GSH/TGSH and ASC/TASC in maize plants compared to their control plants. Interestingly, the measured percentages were enhanced (80%) under treatment of NRS_31 and/or Co, compared to the control plants. Overall, the combined treatment had positively affected the antioxidant defense system, thereby alleviating the oxidative stress impact of Co stress on maize plants.

### Effect of Archaea on Enzymatic Antioxidants

To get more insights into Co and archaea induced effects on the antioxidant defense system of maize plants, we analyzed the changes in ascorbate–glutathione cycle enzymes and metabolites. First, we measured the enzymes that are incorporated in direct scavenging activity, i.e., catalase (CAT) and peroxidase (POX) and superoxide dismutase (SOD). POX, CAT, and SOD showed a remarkable increase in their activities under Co stress compared to their control values. Such increases were much more pronounced (by 220, 150, and 80%, respectively) in NRS_31-treated plants, compared to their respective control plants (Table 4). When combined with each other, strain NRS_31 and Co treatment resulted in dramatic increases in the activities of POX, CAT, and SOD enzymes (by 320, 350, and 120%, respectively). The induction of these enzymes could play a role in the removal of the produced H_2_O_2_. Thus, reduced H_2_O_2_ content of in inculcated maize plants under Co treatment might be explained by increased antioxidant activities of antioxidant enzymes.

Next, we investigated the ascorbate-related enzymes in the ascorbate–glutathione cycle, i.e., ascorbate peroxidase (APX), dehydroascorbate reductase (DHAR), and monodehydroascorbate reductase (MDHAR). The activities of APX and DHAR were increased in response to Co treatment, whereas the activity of MDHAR was not significantly changed (Table 4). Similarly, the treatment with strain NRS_31 induced higher APX, DHAR, and MDHAR activities (220, 70, and 100%, respectively) in comparison with the control plants. The interaction between archaea and Co has further increased the activity of APX, DHAR, and MDHAR enzymes (320, 110, and 120%, respectively) compared to the control.

Further, we measured the activities of GSH-related enzymes [glutathione peroxidase (GPX) and glutathione reductase (GR)] that catalyze the reduction of GSSH and thus maintains GSH in its reduced form. In our study, the GPX activity was increased when maize plants were grown under Co stress, whereas no changes were observed in GR activity. Likewise, the treatment with strain NRS_31 increased the GR and GPX enzymes activities (by 80 and 100%, respectively) compared to control plants. Under the combined treatment of both strain NRS_31 and Co, the maize plants exhibited much higher activities in GR and GPX (by 150 and 250%, respectively) as compared to the corresponding control plants.

Finally, to draw a complete picture about the response of the antioxidant defense system to the impact of Co and/ or strain NRS_31 treatment, we evaluated the activities of glutaredoxins (Grx) and thioredoxin (Trx) which are involved in scavenging of free radicals. Co treatment enhanced the activities of Grx, Trx, and peroxiredoxins as compared to the control. Such enzyme activities were much more increased in plants treated with strain NRS_31, particularly peroxiredoxins (by 250%) in comparison with their respective control plants. The treatment with strain NRS_31, combined with Co treatment, greatly improved the activities of Grx, Trx, and peroxiredoxin enzymes (370, 230, and 400%, respectively) compared to the corresponding control plants. Overall, the activation of antioxidant system was in parallel with the increased levels of ROS.

### Effect of Archaea on Co Detoxification

**Figure 4:**
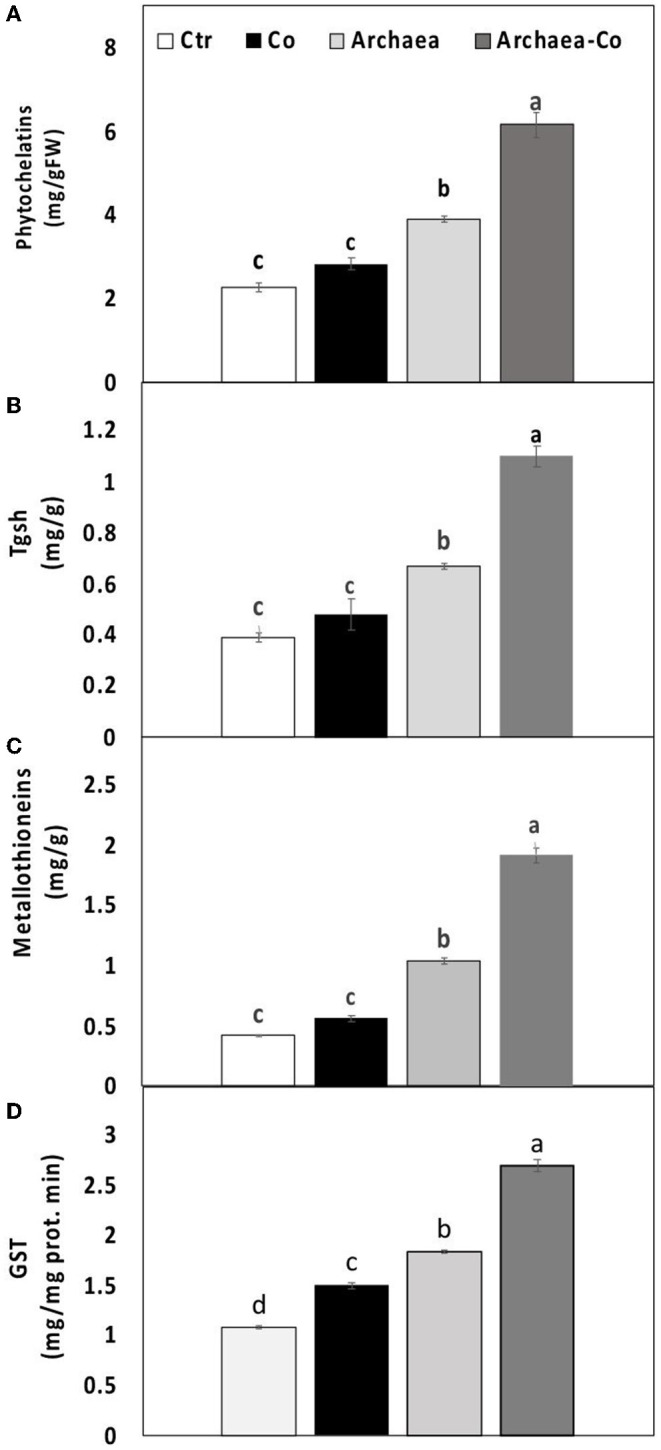
Effect of cobalt (Co) on **(A)** phytochelatins, **(B)** total glutathione (TGSH), **(C)** metallothionein, and **(D)** glutathione S transferase activity (GST) in maize plants inoculated and non-inoculated with strain NRS_31. The mean values and standard errors were calculated based on 3 biological replicates (*p* < 0.05; *n* = 3). The significant changes among the treatments were indicated by the different characters.

To overcome heavy metal toxicity, plants tend to synthesize some heavy metal-binding ligands, such as metallothioneins (MTC), phytochelatins (PCs), and the metal detoxifying enzymes such as TGSH and GST, which were explored in our study (Figure 4). The level of TGSH and the activity of GST were enhanced under Co treatment conditions, but no significant differences were observed in PC and MTC. Under treatment with strain NRS_31, all the measured parameters were increased compared to untreated plants. Interestingly, a combination of strain NRS_31 and Co treatments has dramatically enhanced the contents of PCs and MTC and also the activities of TGSH and GST (200, 400, 250, and 170%, respectively). Thus, the increased activities of metal-binding ligands, particularly under the combined treatment of strain NRS_31 and Co, could contribute to heavy metal detoxification.

## Discussion

Archaea are a large, diversified, and widely distributed microbial community in the geosphere (Karner et al., 2001; Baker et al., 2020). They are the key members of the plant microbiome and can interact with a wide range of plant species (Taffner et al., 2018). Despite the fact that archaea are an important part of the plant microbiome, they have received less attention as potential plant growth-promoting archaea. Here, we investigated the growth-promoting enfeoffment archaea on an economic crop (maize) grown under Co stress circumstances. A better understanding of recent developments in archaea bioactivity and plant-colonizing ability could facilitate their eventual usage as the reliable components of sustainable agricultural systems (Alori et al., 2020). Indeed, the finding that archaea oxidize ammonia in soil and produce growth-promoting metabolites such as IAA diverted the intention toward the use of archaea as growth-promoting microorganisms (Finnie and Van Staden, 1985; Leininger et al., 2006). Aklujkar et al. (2014) and White (1987) reported the production of plant growth-promoting hormones including IAA by archaea such as *Sulfolobus acidocaldarius*. In this study, the selection of strain NRS_31(identified as *Haloferax*) was based on the increased production of IAA, siderophore, and other biochemical characters. Microorganisms might interact with extracellular metal ions and hence contribute to change the physical and chemical properties of soil (Ma et al., 2016; White et al., 2016). In this context, siderophores have been implicated as a factor in archaea's apparent impacts on heavy metal uptake (Yadav et al., 2017). Moreover, some archaea, including *Natrialba*, and *Haloferax* had other important plant growth-promoting attributes such as N fixation and phosphorus solubilization (Yadav et al., 2017).

The natural Co content of soil is up to 40 mg/kg (Blume et al., 2016). Cases of exceeding its permissible content in the soil primarily result from the intensive extraction of this element due to its wide application in various industries. Consequently, Co accumulation in plants significantly inhibits plant growth and yield. On the other hand, the haloarchaeal strain NRS_31 holding inoculum stimulated the growth of maize plant and, interestingly, neutralized the negative growth inhibitory Co effects. Here, soil was first incubated with haloarchaeal species strain NRS_31 for 3 days to ensure archaea growth before adding cobalt. Nonetheless, in other preliminary experiments, both Co and archaea inoculum were mixed in the same day and archaea still showed similar positive effects on maize growth.

Previously, Dave et al. (2006) reported that archaea can produce carboxylate siderophores and sequester iron. The production of organic acids and pH reduction by some strains of archaea favor phosphorus solubilization by archaea. In maize microbes, medicated detoxification of harmful chemicals is reported *via* induction of metal-binding ligands, such as MTC and PC (Elsgaard et al., 2001). Interestingly, in this work, the inoculum containing Co and archaea dramatically boosted the activity of metal detoxifying enzymes such as TGSH and GST in maize. Similar effects were previously observed when *Brassica napus* seedlings were inoculated with bacteria under copper stress. In other study, the bacterium neutralized the growth inhibitory effects of copper stress on copper-treated seedlings (He et al., 2010). Similarly, the inoculation of bacteria increased the tolerance of *Brassica juncea* to the toxicity of lead and zinc (Wu et al., 2006). Thus, our data support the findings that archaea can change heavy metal bioavailability by chelating compounds, modifying the microenvironment, and changing the redox potential (McGrath et al., 2001; Lasat, 2002; Kushwaha et al., 2015).

The observed enhanced biomass production by archaea strain NRS 31 was attributed to a higher rate of photosynthesis, which was consistent with many prior findings for other plants (Jung et al., 2020). Overall, plant–archaeal interactions boosted photosynthesis and strengthened cellular processes which resulted in higher growth and yield. Induced photosynthesis also increased sugar metabolism, allowing more carbohydrates to be allocated to Co stress defense. Here, this was correlated with increased sucrose metabolic enzymes including SPS and invertases enzymes. Creus et al. previously demonstrated that in stress, phosphatidylglycerophosphatase (PGPB) effectively increased *Triticum aestivum* growth and yield *via* water homeostasis and osmolyte accumulation, which included N-acetylated amino acids such as glutamate and proline, peptides, and sugars such as sucrose and trehalose (Creus et al., 2004). In this study, archaea alone or in combination with Co increased sugar (invertase and SPS) and proline biosynthesis (P5CS and P5CR). Both sugar and proline serve as an energy source and organic building blocks, as well as maintaining the osmotic potential of the plant cell, protecting membranes, and stabilizing photosystem II (Sami et al., 2016). Consequently, the accumulation of these compounds in response archaea treatment could be used to maintain high osmotic potential of Co-stressed plants.

Sugars have also a significant capacity for ROS, in addition to their osmoregulation capabilities (Matros et al., 2015). For instance, elevated sugars increased the activity of antioxidant enzymes (Tewari et al., 2002). However, over-accumulation of ROS under stress affects numerous biological processes, such as membrane-bound transport systems and lipid peroxidation (Pandey et al., 2009). In this study, Co treatment induced the oxidative stress in maize, which is revealed by drastic increases in concentration of H_2_O_2_, MDA, protein oxidation, and photorespiration enzymes, i.e., HPR and GO. However, when Co was combined with archaea, there was a slight increase in enzyme activity. Similarly, functional assessments revealed that archaea contain stress defense, which can ameliorate detrimental effects of abiotic stress (Taffner et al., 2019).

Further, in response to heavy metal-induced oxidative damage, plants have evolved various antioxidative defense systems that include both enzymatic and non-enzymatic antioxidant (Tewari et al., 2002; Karuppanapandian et al., 2011). For instance, it has also been reported that the oxidative stress activates the expression of genes responsible for the synthesis of antioxidants in plants (Munné-Bosch, 2005). Here, the plant growth-promoting archaea further increased the antioxidant defense system in Co-treated maize plants. The results are in line with the findings that the growth-promoting bacteria increased plant antioxidant capacity (Nautiyal et al., 2008). Similarly, growth-promoting bacteria induced the synthesis of phenolics at different growth stages of chickpea seedlings (Singh et al., 2003; Kisa et al., 2016). Furthermore, various enzymes, such as APX, CAT, POX, and SOD, involve in amelioration of biotic and abiotic stress were also induced in response to bacterial inoculation (Nivetha et al., 2021). For instance, the inoculation of PGPRs improved salinity stress tolerance in wheat through alleviating the activity of numerous antioxidant enzymes such as manganese-dependent superoxide dismutase (MnSOD), POD, CAT, GR, and APX (Bharti et al., 2013). The findings are in line with this study, where the excess Co significantly increased enzyme activity responsible for dismutation of O_2_, which were further increased by inoculation with archaea (Porteri et al., 2009). Furthermore, our results revealed that the activities of Grx, Trx, and peroxiredoxins, families of thiol oxidoreductases, were also increased, but again, they were higher in archaea combined with Co treatment. In this regard, H_2_O_2_ is known to control the pool of GSH in plants and executes posttranslational regulation of GR transcript levels, thereby controlling GSH synthesis and GR activity (Kellos et al., 2008).

Overall, the research addressed the potential role of archaea to improve sustainable crop production in heavy metal contaminated-soils. More studies should be directed toward a complete understanding of the mechanisms behind plant growth promotion by archaea. The importance of such studies like this is to highlight the metabolic pathways that are specific to certain plant species or groups and are upregulated in response to certain stress factors. Hence, these pathways can be manipulated genetically or through the use of chemical or biological factors to produce crops that are specifically capable of coping with these stress factors.

## Conclusion

Based on the above results, it could be concluded that archaea are able to mitigate the adverse effects of Co stress on plants. This was positively reflected on improving the growth and photosynthesis of maize grown under the combined effect of archaea and Co. The enhanced photosynthetic efficiency improved sugar metabolism. In addition, the antioxidant metabolites and antioxidant enzymes were greatly increased. Overall, the combined treatment with archaea and Co could represent an alternative solution to overcome the heavy metal toxicity to plants. Finally, while archaea may be important for plant growth and development, quite a lot remains to be done to make this possibility into a commercial reality.

## Data Availability Statement

The original contributions presented in the study are included in the article/supplementary material, further inquiries can be directed to the corresponding author/s.

## Author Contributions

SS, HA, AA, MW, EE, ME, MMA, SJ, and NH: research design, data analyses, and funding resources. SS, HA, AA, MW, EE, ME, MMA, SJ, NH, and MA: perform the experiments and writing the first draft. SS, AA, MW, EE, ME, MA, SJ, NH, and AS: data analyses. SS, AA, MW, EE, ME, MA, SJ, and AS: supervision. SS: funding and resources. All authors contributed to the article and approved the submitted version.

## Funding

We extend their appreciation to the Deanship of Scientific Research at Jouf University for funding this work through research (Grant No. DSR-2021-01-0222).

## Conflict of Interest

The authors declare that the research was conducted in the absence of any commercial or financial relationships that could be construed as a potential conflict of interest.

## Publisher's Note

All claims expressed in this article are solely those of the authors and do not necessarily represent those of their affiliated organizations, or those of the publisher, the editors and the reviewers. Any product that may be evaluated in this article, or claim that may be made by its manufacturer, is not guaranteed or endorsed by the publisher.
